# FoxO1 Is a Novel Regulator of 20S Proteasome Subunits Expression and Activity

**DOI:** 10.3389/fcell.2021.625715

**Published:** 2021-02-05

**Authors:** Marianna Kapetanou, Tobias Nespital, Luke S. Tain, Andre Pahl, Linda Partridge, Efstathios S. Gonos

**Affiliations:** ^1^Laboratory of Molecular and Cellular Aging, Institute of Chemical Biology, National Hellenic Research Foundation, Athens, Greece; ^2^Department of Biological Mechanisms of Ageing, Max Planck Institute for Biology of Ageing, Cologne, Germany

**Keywords:** FOXO factors, insulin signaling, proteostasis, proteasome, aging, longevity

## Abstract

Proteostasis collapses during aging resulting, among other things, in the accumulation of damaged and aggregated proteins. The proteasome is the main cellular proteolytic system and plays a fundamental role in the maintenance of protein homeostasis. Our previous work has demonstrated that senescence and aging are related to a decline in proteasome content and activities, while its activation extends lifespan *in vitro* and *in vivo* in various species. However, the mechanisms underlying this age-related decline of proteasome function and the down-regulation in expression of its subunits remain largely unclear. Here, we demonstrate that the Forkhead box-O1 (FoxO1) transcription factor directly regulates the expression of a 20S proteasome catalytic subunit and, hence, proteasome activity. Specifically, we demonstrate that knockout of FoxO1, but not of FoxO3, in mice severely impairs proteasome activity in several tissues, while depletion of IRS1 enhances proteasome function. Importantly, we show that FoxO1 directly binds on the promoter region of the rate-limiting catalytic β5 proteasome subunit to regulate its expression. In summary, this study reveals the direct role of FoxO factors in the regulation of proteasome function and provides new insight into how FoxOs affect proteostasis and, in turn, longevity.

## Introduction

Proteostasis is a pivotal process indispensable for the majority of cellular functions, including DNA replication, the regulation of the cell cycle, metabolism, maintenance of cellular architecture, signaling pathways, development and immune responses (Powers et al., 2009; Labbadia and Morimoto, 2015). Proteostasis collapse has been documented as a key factor contributing to the progression of aging (López-Otín et al., 2013), caused by a gradual failure of the respective defense systems (Taylor and Dillin, 2011). Furthermore, several studies have demonstrated that chronic exposure to aggregated or denatured proteins contributes to the development of age-related diseases, such as Alzheimer’s and Parkinson’s disease (Chondrogianni et al., 2015b; Labbadia and Morimoto, 2015). The proteasome plays a pivotal role in maintaining proteostasis and as such, is involved in a multitude of biological processes (Vilchez et al., 2014). The 30S/26S proteasome is the main proteasome complex consisting of the 19S regulatory “cap” and the 20S catalytic “core” (single capped: 26S, double capped: 30S). The 20S core proteasome has barrel-like configuration and is comprised by seven different α subunits and seven distinct β subunits. Three β subunits, namely β1, β2, and β5, possess proteolytic activities with different substrate specificities (Chondrogianni et al., 2014). Our previous work has established a direct association between proteasome-mediated proteolysis and aging. Specifically, we have demonstrated that the accumulation of damaged proteins during aging is connected to an age-related downregulation of proteasome expression and activity. In addition, pharmacological or genetic induction of the proteasome improves both cellular and organismal lifespan and alleviates the pathological phenotype of protein aggregation-related diseases, such as Alzheimer’s disease (Chondrogianni et al., 2015b; Mladenovic Djordjevic et al., 2021). Moreover, we have shown that human mesenchymal stem cells (hMSCs) exhibit a senescence-related decline of proteasome content and aberrations in physiological assembly of proteasome complexes during prolonged *in vitro* expansion, while proteasome activation via overexpression of the catalytic β5 subunit can enhance their stemness and lifespan (Kapetanou et al., 2017). Therefore, we hypothesize that the mechanisms and molecular factors that control proteasome subunit expression are crucial regulators of longevity. However, the respective underlying mechanisms remain largely obscure.

Numerous studies have identified an extensive array of genes that can alter the lifespan of several organisms. Despite this enormous volume of research, we still do not completely comprehend how these genes influence the aging process of an organism (Flatt and Partridge, 2018). A proteomic study in *D. melanogaster* (Tain et al., 2017) and a genetic approach in *C. elegans* (Vilchez et al., 2012) have suggested a potential interplay between the regulation of the proteasome and the Forkhead box-O (FoxO) transcription factors. FoxO factors control several cellular processes like autophagy and apoptosis in response to signals emanating from the environment and are important longevity determinants, downstream of insulin and insulin-like growth factor signaling (IIS). FoxO factors, under conditions of low IIS, translocate into the nucleus and bind to promoters of pro-longevity genes to regulate transcription. Nevertheless, their complex role in life-expectancy determination has not been fully elucidated yet. Here, we have dissected further the mechanism of IIS action on mammalian proteasome regulation and demonstrate for the first time that FoxO1 directly regulates the expression and activity of the 20S proteasome.

## Materials and Methods

### Mouse Models and Husbandry

All mice were maintained at 22°C under a 12-h light/dark cycle (lights on from 7:00 am to 7:00 pm). Mice were housed in groups of three to five same-sex littermates under specific pathogen-free conditions within individually ventilated cages (Tecniplast UK Ltd., Kettering, Northamptonshire, United Kingdom). Mice had *ad libitum* access to normal chow [ssniff^®^ R/M-H phytoestrogen-poor (9% fat, 34% protein, and 57% carbohydrate) ssniff Spezialdiäten GmbH, Soest, Germany] and water. *Irs1* global knockout mice were generated as described previously (Selman et al., 2008). The conditional FoxO1 (from Ron DePinho, MD Anderson Cancer Center) total knockouts were induced by tamoxifen treatment for 6 weeks using a ROSA26-CreERT2-mediated recombination (Supplementary Figure 1, mouse line from Thomas Langer, MPI for Biology of Aging) (Paik et al., 2007). *Foxo1* (*n* = 8, 6 females and 2 males) and *Irs1* (*n* = 7, females) KO and their littermate controls (+/+, *n* = 7, 5 females and 2 males and *n* = 7, females, respectively) were sacrificed at 16 weeks. *Foxo3* total knockouts were generated by using germline Cre-mediated recombination (actin-Cre) with the conditional *Foxo3* allele (from Ron DePinho, MD Anderson Cancer Center) producing the null allele. Mice were dissected and tissues were snap-frozen in liquid nitrogen.

### Mouse Embryonic Fibroblast Isolation and Culture

Primary mouse embryonic fibroblasts (MEFs) were isolated from *Irs1*+/+ or *Irs1*−/− animals and cultured according to standard procedures (Qiu et al., 2016). Briefly, MEFs were maintained in Dulbecco’s modified Eagle’s medium (DMEM; Invitrogen) supplemented with 10% fetal bovine serum (v/v; Invitrogen), 2 mM glutamine and 1% non-essential amino-acids at 37°C, 5% CO_2_ and 95% humidity.

### Reagents and Antibodies

LLVY-AMC, MG132 and the primary antibody against the proteasomal subunits β5 (X, MB1, ϵ; PW8895; 22.9 kDa) and α6 (C2; PW8100; 33 kDa) were purchased from Enzo Life Sciences, Inc. The ChIP Grade antibody against FoxO1 (ab39670) and HRP-conjugated anti-rabbit and anti-mouse antibodies were purchased from Abcam.

### Proteasome Peptidase Assays

Liver and brain tissues were lysed in 25 mM Tris/HCl lysis buffer, pH 7.6 containing 5 mM ATP, 10% glycerol, 20 mM KCL, 1 mM EDTA, 1 mM DTT, 0.2% Nonidet P-40, 10 mM phenylmethylsulfonyl-fluoride and 10 μg/ml aprotinin (Rivett et al., 1994). The CT-L activity of the proteasome was assayed after the incubation of 10 μg of total protein for 30 min at 37°C with the fluorogenic peptide LLVY-AMC, as previously described (Georgila et al., 2014). Proteasome activity was determined as the difference between total fluoresce and fluoresce in the presence of 20 μM of the proteasomal inhibitor MG132. AMC fluorescence was measured at 360 nM excitation and 460 nM emission using a spectrofluorimeter (Tecan). A Bradford assay was used to determine protein concentration, using bovine serum albumin as standard.

### Immunoblot Analysis

Twenty μg protein of isolated protein were separated by 10% SDS-PAGE under non-reducing conditions according to standard procedures (Palmer, 2000). Following electrophoresis, protein loading was analyzed using the Stain-free^TM^ (Bio-Rad) imaging technology that allows the visualization of the proteins directly in the gel after a short photoactivation. Proteins were then transferred to nitrocellulose membrane (Amersham Biosciences) to be treated with the blocking buffer and were subsequently incubated with the appropriate antibodies. The primary antibodies were detected with horseradish peroxidase conjugated secondary antibodies. The detection with enhanced chemiluminescence was performed using ECL or ECL prime chemiluminescence kits (GE Healthcare) and a ChemiDoc station (Bio-Rad).

### Real Time PCR Analysis

For the characterization of proteasome genes, total RNA was isolated using TRIzol (Invitrogen) and transcripted into cDNA with the cDNA iScript synthesis kit (Bio-Rad). The Real time PCR were run on the CFX Connect Real-Time PCR System (Bio-Rad). The RT-PCR primers are summarized in Supplementary Table 1. For the evaluation of FoxO1 knockout efficiency, RNA was isolated using TRIzol (Thermo-Fisher), treated with DNAse (Qiagen) and purified by isopropanol precipitation. cDNA was prepared using the SuperScript III reverse transcriptase kit (Invitrogen) as per manufacturer’s instructions. TaqMan probes against Foxo1 and beta2-microglobulin were obtained from Applied Biosystems and run on a 7900HT real-time PCR system.

### ChIP Analysis

Chromatin immune precipitation experiments were performed using the ChIP-IT^®^ Express Enzymatic kit (Activemotif, cat. no 53009), as per manufacturer’s instructions. Chromatin was sheared enzymatically for 5 min and precipitated with a ChIP-Grade Anti-FOXO1 antibody (ab39670, Abcam). Prior to amplification, the samples were subjected to DNA clean-up step using the Nucleospin R Gel and PCR Clean-up kit (740609.10, Macherey-Nagel). The ChIP products were then analyzed by Real-time PCR and the products were confirmed by agarose gel electrophoresis. An anti-IgG antibody was used as a negative control.

### RNA Interference

The small interfering (si)RNAs targeting murine FoxO1 and IRS1 were obtained from Thermo Fisher Scientific (s80620) and Sigma (EMU061331 MISSION^®^ esiRNA), respectively. The siRNA targeting murine FoxO3 has been purchased from Thermo Fisher Scientific (Silencer^®^, 100380). Briefly, the siRNA duplexes were transfected into 70–80% confluent MEFs cultured in a in a 6-well or 96-well plate format at a final concentration of 50 nM, in presence of the TransFectinTM Lipid Reagent (1703351, Bio-Rad) at a ratio of 1:2. Transfection complexes were prepared in Opti-MEM^®^ Reduced Serum Medium, GlutaMAX^TM^ Supplement (51985-034, Thermo Fisher Scientific). After 24 h, the cells were harvested or transfected with the LightSwitch^TM^ plasmids without media change.

### FoxO1 Activity Assay

The isolation of nuclear proteins and the subsequent examination of FOXO1 transcriptional activity were performed with the Nuclear Extraction Kit (ab113474, Abcam) and the FOXO1 Transcription Factor Assay Kit (Colorimetric; ab207204, Abcam), respectively, according to manufacturer’s instructions.

### Luciferase Assay

The promoter region of β5 and a mutagenized version (Supplementary Table 2) were cloned into the LightSwitch^TM^ Promoter Reporter Vector (Active Motif). MEFs were transfected using the Transfectin reagent (1703351, Bio-Rad) and the luciferase assay was performed with the LightSwitch^TM^ Luciferase Assay Kit (32031, Active Motif) in quadruplicates, according to manufacturer’s instructions. Each well was read for 10 s and the signal of the empty vector (32021, Active Motif) was deducted from the values of sample wells.

### Ethics Statement

This study was performed in strict accordance with the recommendations and guidelines of the Federation of European Laboratory Animal Science Associations (FELASA). The protocol was approved by the Landesamt für Natur, Umwelt und Verbraucherschutz Nordrhein-Westfalen.

### *In silico* Identification of FoxO Binding Motifs

Genomic DNA sequences were downloaded in FASTA format from www.ensembl.org/ and individual matches of known FoxO binding motifs were scanned using FIMO^1^ in 1 kb regions upstream of the TSS of murine 20S proteasome genes.

### Statistical Analysis and Quantifications

Statistical analysis and the graphical representation of data was performed using the GraphPad Prism 5 (GraphPad Software, San Diego, CA, United States). All values were reported as mean ± SE, unless otherwise indicated. Densitometry analysis for the quantification of immunoblots was performed with Bio-Rad’s Image Lab software 6.0.1. The average signal of control mice was arbitrarily set to 100% or 1.

## Results

### Foxo1 Knockout Mice Exhibit Reduced Proteasome Activity and Expression in Liver and Brain

**FIGURE 1:**
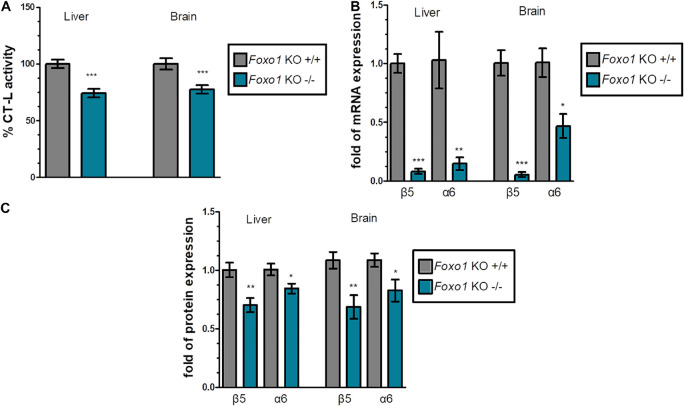
FoxO1 depletion downregulates the proteasome: **(A)** Mean% CT-L activities, **(B)** mRNA expression, and **(C)** Mean values immunoblot analysis of the indicated proteasome subunits in the liver and brain of the indicated control and FoxO1-depleted mice. The total protein load was used as a control for equal protein loading and GAPDH was used for RT-PCR normalization. 100% or 1 has been arbitrarily set to the average values of the control samples. Data information: *P < 0.05, **P < 0.01, ***P < 0.001. All error bars show SEM. Number of animals: *Foxo1*^–/–^ *n* = 8, 6 females and 2 males and *Foxo1*^+/+^ *n* = 7, 5 females and 2 males.

Firstly, we evaluated the proteasome status in various tissues of *Foxo1* KO mice sacrificed at 16 weeks of age, in comparison to their respective control animals. We found that *Foxo1* mutant mice displayed a substantial decrease in chymotrypsin like (CT-L) activity. Specifically, the data for liver and brain that are shown in Figure 1A and Supplementary Figures 2A,3A revealed a statistically significant reduction of proteasome activity by 25.8% and 22.6% in liver and brain, respectively. In support, the mRNA (Figure 1B) and protein levels (Figure 1C and Supplementary Figures 2B,3B) of representative β and α 20S subunits (catalytic β5 and α6) declined considerably in absence of FoxO1 in these tissues. However, FoxO3 depletion affected neither proteasome expression nor its activity (Supplementary Figure 6).

### Irs1 Global Knockout Enhances Proteasome Expression and Function

**FIGURE 2:**
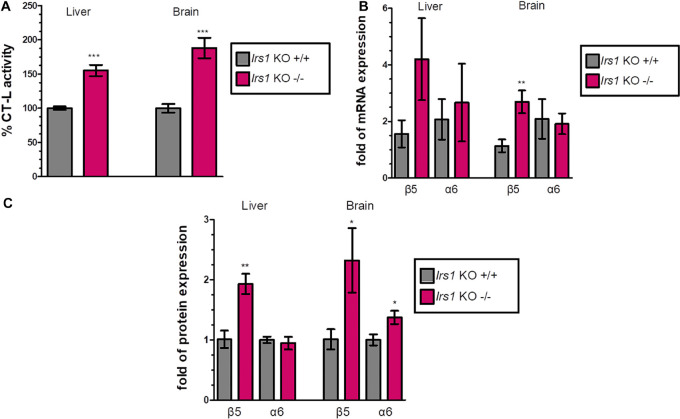
Global *Irs1* knockdown induces the proteasome: **(A)** Mean% CT-L activities, **(B)** mRNA expression and **(C)** Mean values immunoblot analysis of β5 and a6 proteasome subunits, in the liver and brain of the indicated control and IRS1-depleted mice. The total protein load was used as a control for equal protein loading and GAPDH was used for RT-PCR normalization. 100% or 1 has been arbitrarily set to the average values of the control samples. Data information: *P < 0.05, **P < 0.01, ***P < 0.001. All error bars show SEM. Number of animals: *Irs1*^–/–^ *n* = 7, females and *Irs1*^+/+^, *n* = 7, 5 females and 2 males.

FoxO factors are activated when IIS activity is low to induce the expression of their target genes. To examine the effect of FoxO activation on the proteasome-mediated proteolysis, we characterized the proteasome status of the long-lived *Irs1* KO mice (sacrificed at 16 weeks of age) and observed that the CT-L activity was increased (see, Figure 2A and Supplementary Figures 4A,5A) by 55.3% in the liver and by 88.2% in the brain. Additionally, there was a consistent pattern of induced mRNA (Figure 2B) and protein expression (Figure 2C and Supplementary Figures 4B,5B) of the β5 subunit in *Irs1* KO mice in comparison to their relative control littermates. However, the expression of α6 was not consistently upregulated by *irs1* knockdown, suggesting that IIS has a more prominent role in the regulation of β5.

### FoxO1 Directly Binds to the Murine Promoter Region of β5

**FIGURE 3:**
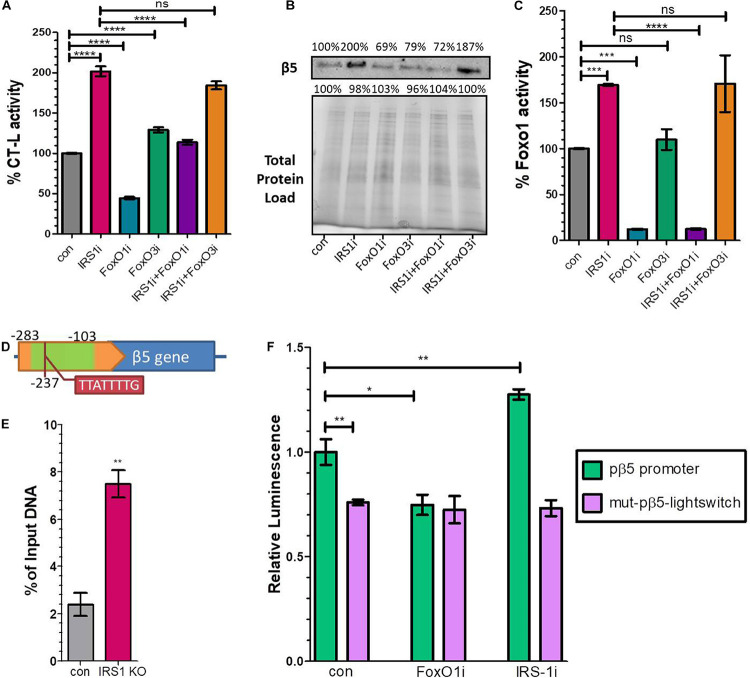
FoxO1 binds to the murine β5 promoter to regulate gene expression. **(A)** % CT-L activities, **(B)** Immunoblot analysis of the β5 subunit and **(C)** FoxO1 activity in the presence of siRNAs targeting IRS1, FoxO1, FoxO3 or control as indicated. 100% has been arbitrarily set to the average values of the control samples and the total protein load was used as a control for equal protein loading. **(D)** Schematic diagram of the promoter region of β5 subunit. The green area indicates the amplified chromatin immunoprecipitation products, while the tab indicates the speculatory FoxO binding site. **(E)** Chromatin immunoprecipitation analysis of the β5 promoter region from control or *Irs1* depleted MEFs, using an anti-FoxO1 antibody. The amount of immunoprecipitated DNA was evaluated by RT-PCR. Histogram was constructed by the ratios of the immunoprecipitated DNA to the input (arbitrarily set to 100%). **(F)** Luciferase signal driven by the proximal wild type β5 promoter region (pβ*5-*lightswitch) or by a mutagenized version (mut-pβ5-lightswitch) with 8 bp substitutions on the detected FoxO binding motif in the presence of siRNAs targeting FoxO1, IRS1 or control as indicated. The signal of the empty vector has been deducted from raw values. The luminescence of wild type promoter under control conditions has been arbitrarily set to 1. *P < 0.05, **P < 0.01, ***P < 0.001, ****P < 0.0001.

Our data indicated that the expression of 20S proteasome subunits is positively regulated by FoxO1. To shed light on these observations, we performed IRS1, FoxO1, FoxO3 and double IRS1 + FoxO1 and IRS1 + FoxO3 silencing assays, using small interfering RNAs and tested the CT-L proteasome activity and the levels of the relevant β5 subunit. In support to the described *in vivo* data, IRS1 silencing led to doubled CT-L activity (Figure 3A) as well as to significantly increased β5 levels (Figure 3B), while FoxO1 silencing led to a downregulation of both proteasome activity and β5 content. In addition, the beneficial effects of lowered IRS1 levels on the proteasome were mediated by FoxO1 as double IRS1 + FoxO1 silencing abolished the increase in CT-L activity and β5 expression. In contrast, we observed similar effects of IRS1 and double IRS1 + FoxO3 silencing on proteasome status. Notably, we confirm that IRS1 silencing significantly enhances FoxO1 transcriptional activity (Figure 3C).

To determine if proteasome subunit promoters are occupied by FoxO1 *in vivo*, we analyzed several putative binding sites of FoxO1 containing its consensus sequences 5′-TT[G/A]TTTTG-3′ (Insulin Response Element, IRE) or 5′-TT(G/A)TTTAC-3′ (Daf-16 family binding element, DBE) (Furuyama et al., 2000) that were *in silico* identified in the promoter regions of β1, β2, β5, and β7 subunits, using chromatin IP. Specifically, we examined putative binding motifs that were localized at −374, −577, −237, and −770 upstream of the Transcription Starting Site (TSS) of each gene, respectively. The precipitated fragments were detected using quantitative real-time PCR primers that specifically amplify DNA encompassing the putative FoxO1 binding sites (Supplementary Table 1). As demonstrated in Figure 3D a putative IRE-containing site at −237 upstream of the β5 TSS promoter was detected in FoxO1 immunoprecipitants, using a primer pair that amplified a 180 bp region starting at −281 (Other indicated proteasome subunits promoters were not amplified; data not shown). The non-specific IgG antibody failed to precipitate *in vivo* the proteins bound to this sequence, suggesting that mouse FoxO1 has the potential to bind to the promoter of β5. Specifically, the ChIP-to-Input ratio was 2.4% in control MEFs, suggesting that FoxO1 may be an important factor participating in the regulation of gene expression (Figure 3E). Importantly, there was a 3-fold increase in PCR detection of the FoxO1 binding site in *Irs1* KO MEFs, demonstrating that low IIS activity enhances FoxO1 binding to the promoter of β5 to regulate gene expression.

To assess the functional consequences of FoxO1 binding on the putative IRE (5′-TTATTTTG-3′), the wild type β5 promoter region (−1,000 to 0 upstream the β5 TSS) or a mutagenized version with 8 bp substitutions on the detected FoxO binding motif at −237 were cloned into the pLightSwitch_Prom reporter vector, which utilizes the RenSP luciferase gene (see Supplementary Table 2). As shown in Figure 3F, the mutation on the putative IRE (mut-pβ5-lightswitch) significantly repressed luciferase activity by 26.3% compared to the wild type (pβ*5*-lightswitch), indicating that the respective sequence promotes transcription. Furthermore, co-transfection of siRNAs targeting FoxO1 markedly attenuated the reported activity of the pβ*5-*lightswitch construct by 25.2%, while IRS1 silencing yielded a 27.5% induction of the luciferase activity. Mutation of the detected IRE completely abolished the effects of FoxO1 or IRS1 silencing on promoter activity. These results indicate that the identified site is essential for the FoxO1-mediated regulation of β5 subunit expression.

## Discussion

FoxO transcription factors are conserved regulators of longevity downstream of insulin and insulin-like growth factor signaling. They integrate signals emanating from nutrient deprivation and stress stimuli to coordinate programs of genes involved in cellular metabolism and quality control. The evolutionary conserved function of reduced IIS in organismal lifespan extension has fueled research to understand the mechanisms underlying this pro-longevity function of FoxOs (Webb and Brunet, 2014; Fontana and Partridge, 2015; Martins et al., 2016). However, we still do not fully understand how these factors affect lifespan. In this study, we dissect the contribution of FoxO transcription factors in longevity and demonstrate for the first time using mouse models the ability of reduced IIS to promote 20S proteasome function in mammalian tissues.

The gradual age-related decline of proteostasis maintenance is considered to be an important hallmark of aging (López-Otín et al., 2013). Emerging evidence from various biological systems indicate that FoxOs orchestrate the expression of genes involved in the proteostasis network (Webb and Brunet, 2014). These findings suggest that the maintenance of proteostasis may in part underlie the ability of FoxOs to extend lifespan and to delay signs of age-related diseases. Specifically it has been found that FoxO factors promote the expression of genes involved in autophagy and the ubiquitin–proteasome system. Whereas autophagy is thought to be relatively specific to long-lived proteins and degradation under chronic starvation conditions (Mizushima and Klionsky, 2007), the proteasome system is responsible for the degradation of most short-lived and regulatory proteins (Löw, 2011). Proteasome activity declines during aging in several tissues, including brain, heart, liver, muscle and skin, accounting for the observed accumulation of damaged proteins and the inclusion bodies (Chondrogianni et al., 2015b). Conversely, an intact proteasome is correlated with extreme longevity in humans (Chondrogianni et al., 2000), while proteasome activation can increase cellular and organismal lifespan, alleviate aggregation-related pathologies and enhance stemness (Chondrogianni et al., 2000; Kapeta et al., 2010; Chondrogianni et al., 2015a; Kapetanou et al., 2017). Interestingly, studies in brain have demonstrated the link between proteasome activity and aging (Triplett et al., 2015; Kelmer Sacramento et al., 2020), while Tropea and co-workers have proposed the involvement of IIS in these processes (Wrigley et al., 2017). Other studies on skeletal muscle atrophy have addressed the role of mammalian FoxOs in the positive regulation of ubiquitin ligases. FoxO3 is a strong and direct regulator of the muscle-specific E3 ubiquitin ligases atrogin-1 and Murf-1 transcription (Sandri et al., 2004, 2006; Stitt et al., 2004). In addition to acting upstream of ubiquitination, emerging evidence suggests that FoxO factors are linked to the regulation of proteasome assembly by modulating the expression of a 19S subunit. In human embryonic stem cells (hESCs) and induced pluripotent stem cells, FoxO4 is both necessary and sufficient for expression of the 19S regulatory cap subunit PSMD11 (Rpn6) (Vilchez et al., 2012). The high proteasome activity that ESCs exhibit is considered to be critical for the prevention of senescence. Upon differentiation, PSMD11 expression declines as it is no longer under FoxO4 regulation. This is accompanied by a reduction of proteasome activity and an increase in the levels of polyubiquitinated proteins. Interestingly, FoxOs appear to have a conserved role in regulating proteasome activity. In *C. elegans*, FoxO/DAF-16 promotes various types of stress resistance via the activation of the PSMD11 ortholog, rpn-6 (Vilchez et al., 2012). Similarly, worms overexpressing pbs-5, the ortholog of the catalytic β5 subunit, display a daf-16 dependent increase in lifespan and resistance to proteotoxicity (Chondrogianni et al., 2015a).

However, the mechanisms underlying the exact role of FoxOs in the regulation of 20S proteasome expression and activity remained elusive. In different cell types, FoxO1 and FoxO3 factors modulate various cellular activities, while also having functional redundancies. Herein, we demonstrate that the depletion of FoxO1, but not of FoxO3, leads to a significant decline of proteasome activity in two murine tissues. Notably, the proteasome is an integral part of the cellular function and its inhibition above the observed levels is toxic to cells and tissues, while knockdown of distinct proteasome subunits is embryonic lethal (Tanaka, 2009). The detected reduction of CT-L activity is linked to the reduced mRNA and protein expression of 20S subunits, including the catalytic β5 subunit. Contrariwise, the knockdown of IRS1, which enhances FoxO1 activity and ameliorates lifespan (Taguchi et al., 2007; Kappeler et al., 2008), induces proteasome function and 20S subunits expression. In support, IIS reduction in the fly resulted in an enhanced proteasome assembly and activity in the gut accompanied by a reduction in the aberrant age-related accumulation of proteasome substrates and an increase in gut integrity with age (Tain et al., 2017). Proteasome activity was necessary for IIS-mediated longevity as treatment with low concentrations of a proteasome inhibitor abolished the beneficial effects of IIS reduction in lifespan and gut integrity. Interestingly, proteasome activation was sufficient to increase gut integrity and lifespan in *D. melanogaster*. Likewise, proteasomal inhibition abolished the beneficial effect of reduced IIS on the circuit function in old flies (Augustin et al., 2018).

Furthermore, we demonstrate that silencing of IRS1 leads to enhancement of FoxO1 transcriptional activity, which is in accordance with other studies in mice showing that IRS1 and IRS2 knockout prevents the repressive FoxO1 phosphorylation (Taniguchi et al., 2005; Dong et al., 2006; Cheng et al., 2009; Qi et al., 2013). Importantly, we demonstrate that FoxO1 mediates the beneficial effects of IRS1 downregulation on proteasome CT-L activity and on the protein expression of β5. Supporting the notion that FoxOs may serve discrete or tissue-specific functions (Paik et al., 2007), FoxO3 repression did not downregulate proteasome activity and was not required for the enhanced proteasome activity under conditions of diminished IRS1 expression. These results indicate that FoxO1 is a potent regulator of proteasome function downstream of IIS. FoxO transcription factors target either a conserved DNA binding sequence, 5′-TT(G/A)TTTAC-3′ (daf-16 family binding element, DBE) or the insulin response element (IRE), 5′-TT[G/A]TTTTG-3′ in the promoter regions of their target genes and subsequently regulate gene expression (Furuyama et al., 2000). Importantly, after analyzing putative FoxO1 binding sites in several 20S proteasome subunits, we show that FoxO1 directly binds to the promoter region of β5, in a region containing a candidate IRE element (5′-TTATTTTG -3′). As expected, FoxO1 binding to the β5 promoter was found increased in MEFs lacking IRS1. The functionality of the binding site detected through the ChIP-based experiment was further analyzed by cloning the wild type or a mutagenized β5 promoter into a luciferase reporter system. Supporting the notion that FoxO1 upregulates β5 expression, silencing of FoxO1 led to a significant decrease of β5 promoter activity, while IRS1 silencing led to an increase. Furthermore, the site specific mutagenesis of 8 bp of the detected IRE at position −237 downregulated gene expression and completely blunted the response to FoxO1 or to IRS1 silencing. Notably, mutation of the identified FoxO binding sequence yielded a reduction of luciferase signal similar to FoxO1 silencing. The detected variations of luciferase activity due to mutagenesis or silencing are biologically significant and fall within the typically observed range (Xiong et al., 2012; Singh et al., 2017). Moreover, as β5 is also regulated by other transcriptional factors such Nrf1 (Li et al., 2018), we did not expect that the mutation of the FoxO1 binding site would totally abolish β5 expression. Importantly, β5 in addition to being responsible for the rate-limiting chymotrypsin-like proteolytic activity of the proteasome (Kisselev et al., 1999), was shown to be sufficient to induce the expression of additional proteasome subunits and consequently to enhance 26/30S proteasome assembly and activity when overexpressed in human fibroblasts (Chondrogianni et al., 2005), in human mesenchymal stem cells (Kapetanou et al., 2017) and in *C. elegans* (Chondrogianni et al., 2015a). Hence, the stimulation of the β5 subunit by Foxo1 has implications on the regulation of the whole proteasome machinery. Collectively, these data demonstrate that FoxO1 binds to a functional IRE at β5 promoter to activate gene expression and enhance proteasome activity.

Taken together these data demonstrate that IIS in mice regulates proteostasis and, in turn, lifespan. Our findings provide new insights about the mechanisms regulating the activity of the proteasome and expand our knowledge of how the nutrient signaling pathways affect proteostasis and ultimately longevity. Understanding the mechanisms that mediate the beneficial effects of reduced IIS activity is of major importance for the development of effective treatments to improve health span in humans.

## Data Availability Statement

The original contributions presented in the study are included in the article/Supplementary Material, further inquiries can be directed to the corresponding author/s.

## Ethics Statement

The animal study was reviewed and approved by the Landesamt für Natur, Umwelt und Verbraucherschutz Nordrhein-Westfalen.

## Author Contributions

MK: conceptualization, methodology, data collection and analysis, and writing the manuscript. TN: methodology, data collection and analysis, and editing the manuscript. LT: methodology and editing the manuscript. AP: data collection and analysis. LP: conceptualization, supervision, editing the manuscript, and funding acquisition. EG: conceptualization, supervision, writing and editing the manuscript, and funding acquisition. All authors have read and approved the manuscript.

## Conflict of Interest

The authors declare that the research was conducted in the absence of any commercial or financial relationships that could be construed as a potential conflict of interest.

## Footnotes

1 http://meme-suite.org/tools/fimo

## Abbreviations

ChIP: chromatin immune precipitation
DBE: Daf-16 family binding element
FoxO: forkhead box-O
IIS: *insulin*/IGF-1 *signaling*
IRE: insulin response element
IRS1: insulin receptor substrate 1
KO: knockout
MEFs: mouse embryonic fibroblasts
RNAi: RNA inhibition
RT-PCR: real time polymerase chain reaction
TSS: transcription starting site.
