# Supplementary material for: FoxO1 Is a Novel Regulator of 20S Proteasome Subunits Expression and Activity
Source: Front Cell Dev Biol. 2021 Feb 5;9:625715. doi: 10.3389/fcell.2021.625715 (PMC7901890; doi:10.3389/fcell.2021.625715)
Supplement: Supplementary file 1 [file Data_Sheet_1.DOCX]

Supplementary Material


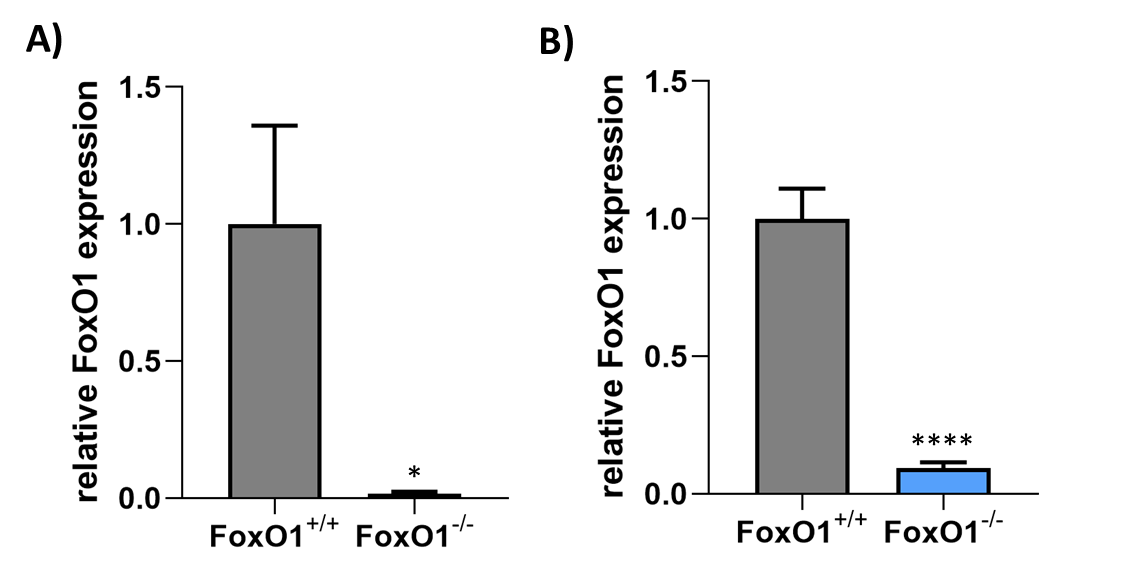


**Figure S1: FoxO1 deletion efficiency through tamoxifen treatment in mouse livers.**  **(A)** Administration of 400 mg/kg tamoxifen for 6 weeks with the chow. q-RT-PCR of FoxO1 transcripts in livers of Cre::Foxo1fl/fl (n = 5) compared to controls (Foxo1fl/fl without Cre, n=4). FoxO1 deletion was induced by this treatment in the following mice: TNES-1800, TNES-1801, TNES-1810, TNES-1813, TNES-1814, TNES-1649, TNES-1696, TNES-1698, TNES-1711,TNES-1712 and TNES-1713. **(B)** Administration of 150 µl tamoxifen solution (10 mg / ml in corn oil) for 10 days via intraperitoneal injection. q-RT-PCR of FoxO1 transcripts in livers of Cre::Foxo1fl/fl (n = 5) compared to controls (Foxo1fl/fl without Cre, n = 4). FoxO1 deletion was induced by this treatment in the following mice: TNES-1805, TNES-1809, TNES-1545, TNES-1706 and TNES-1759. FoxO1 RNA levels are reduced by more than 98 % when administered with the chow and by more than 90 % when injected.* p< 0.05 and **** p< 0.0001 two-tailed unpaired t-test.


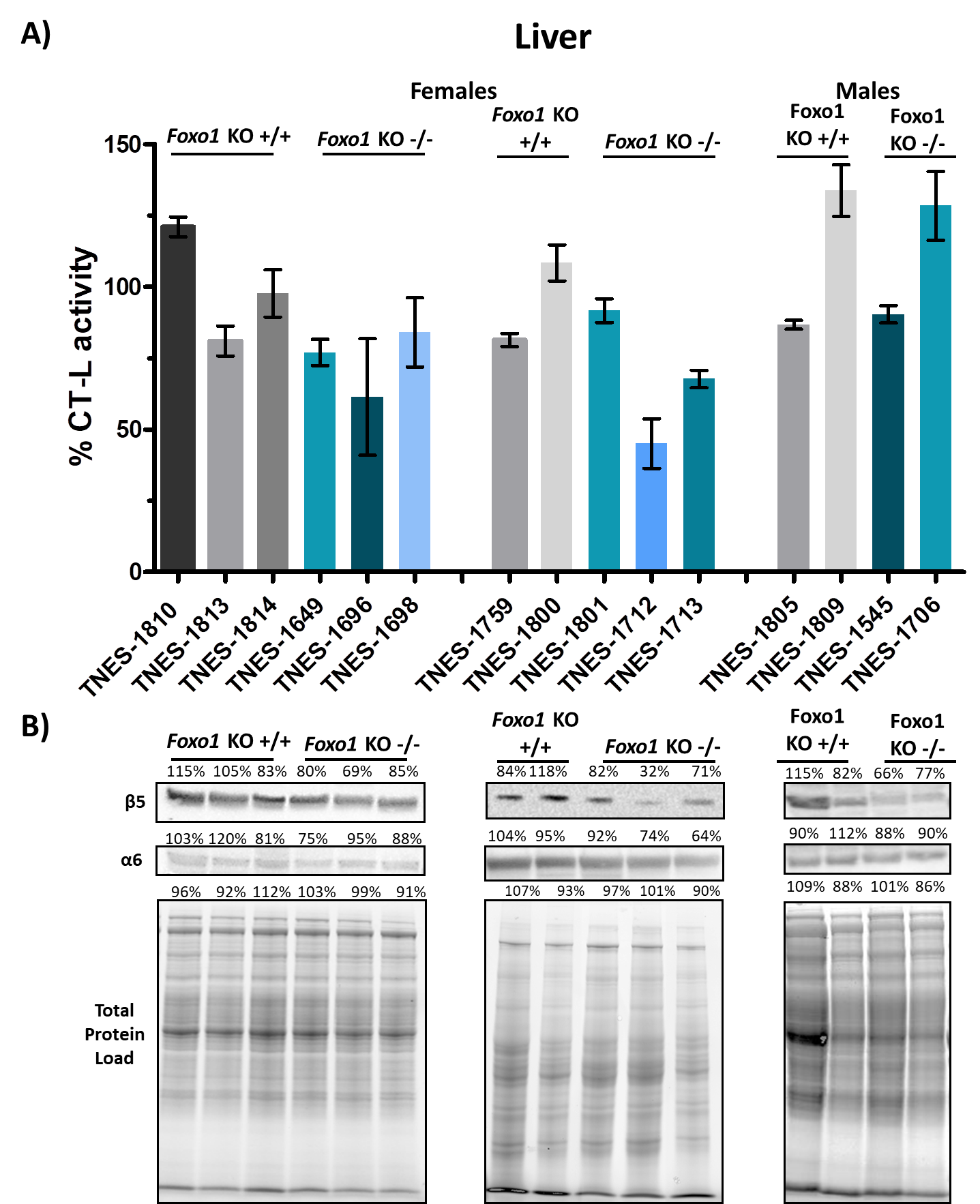


**Figure S2. FoxO1 depletion downregulates the proteasome in murine liver:** (A) % CT-L activities and (B) Immunoblot analysis of β5 (22.9 kDa) and α6 (33 kDa) proteasome subunits in the liver of the indicated control (*foxo1* KO +/+) and FoxO1-depleted mice (f*oxo1* KO -/-). The total protein load was used as a control for equal protein loading. 100% has been arbitrarily set to the average values of each set of control samples. All error bars show SEM.


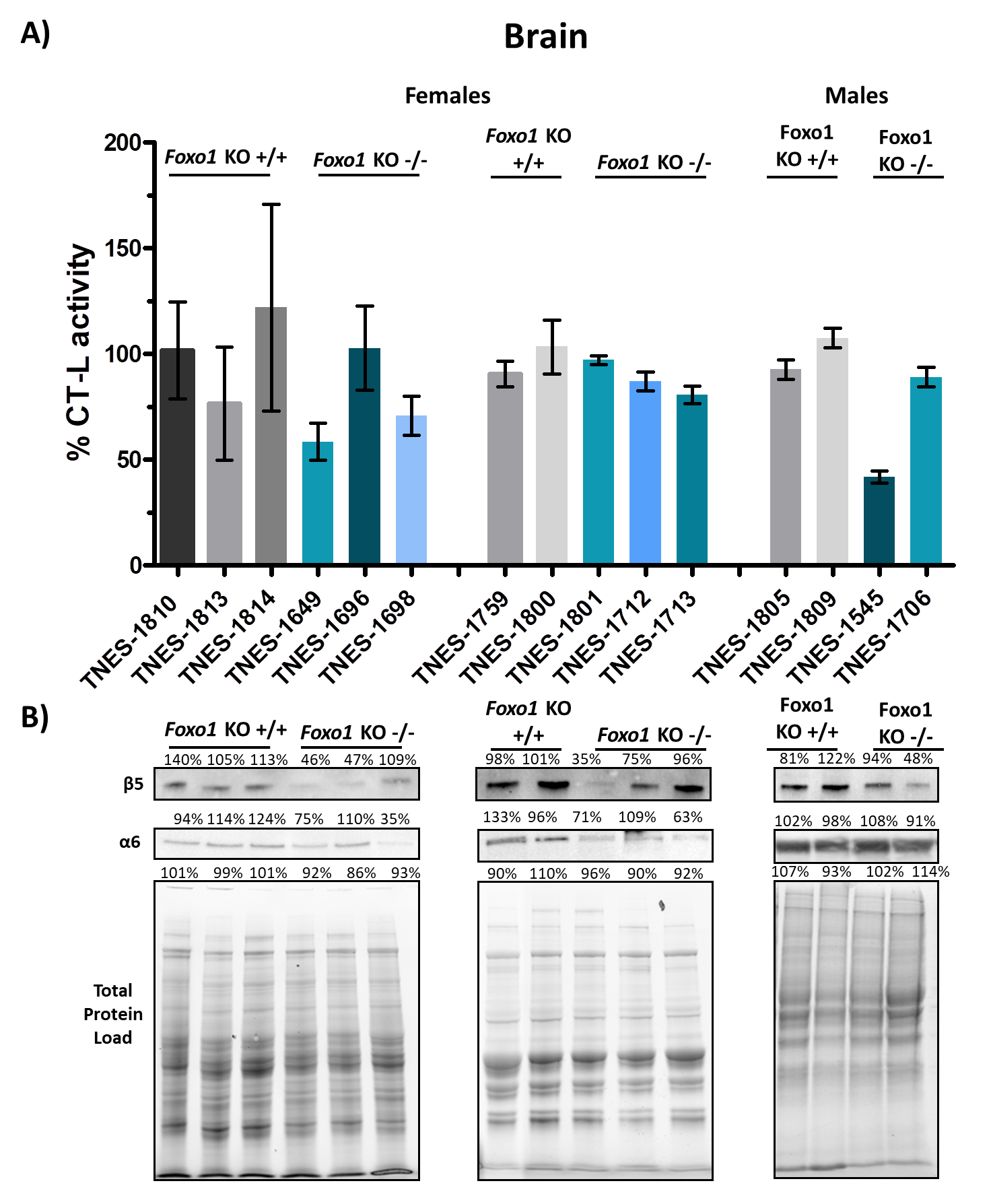


**Figure S3. *FoxO1* knockdown reduces proteasome activity and content in the brain:** (A) % CT-L activities and (B) Immunoblot analysis of β5 (22.9 kDa) and α6 (33 kDa) proteasome subunits in the brain of the indicated control (f*oxo1* KO +/+) and FoxO1-depleted mice (f*oxo1* KO -/-). The total protein load was used as a control for equal protein loading. 100% has been arbitrarily set to the average values of each set of control samples. All error bars show SEM.


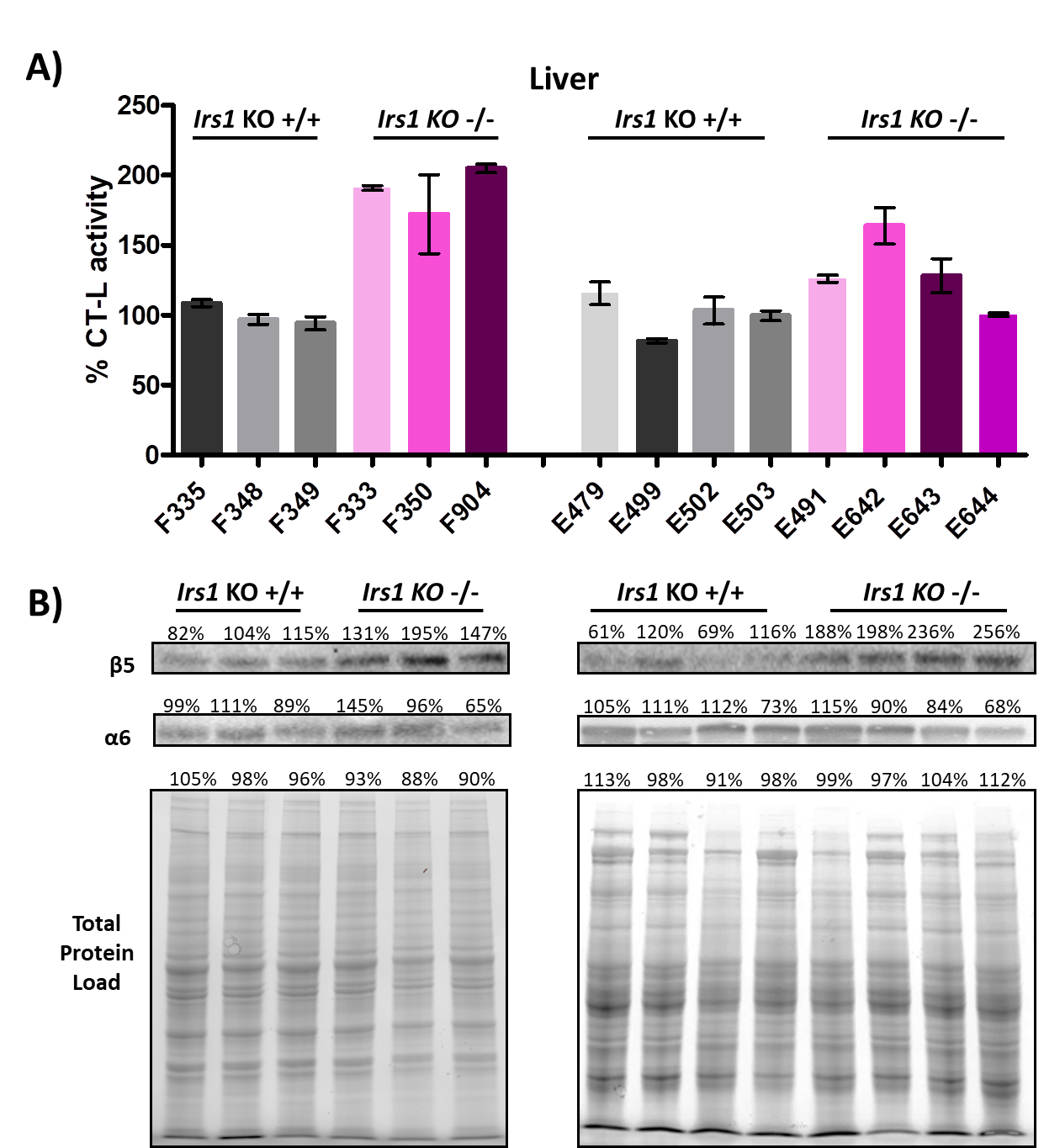


**Figure S4. *Global irs1* knockdown upregulates the proteasome in the liver:** (A) % CT-L activities and (B) Immunoblot analysis of β5 (22.9 kDa) and α6 (33 kDa) proteasome subunits in the liver, of the indicated control (irs*1* KO +/+) and IRS1-depleted mice (*irs1* KO -/-). The total protein load was used as a control for equal protein loading. 100% has been arbitrarily set to the average values of each set of control samples. All error bars show SEM.


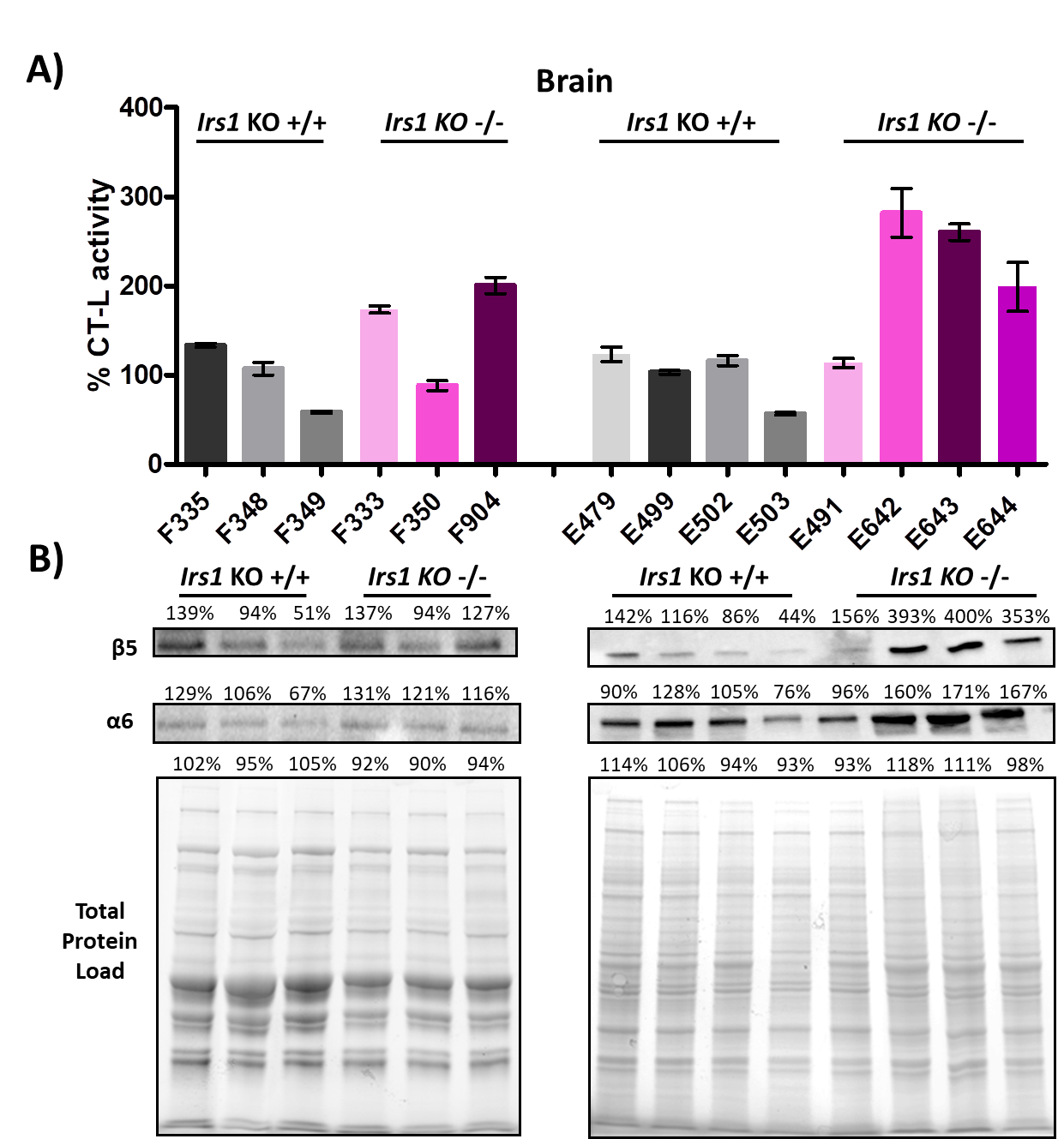


**Figure S5. *Global irs1* knockdown enhances proteasome activity and content in murine brain:** (A) % CT-L activities and (B) Immunoblot analysis of β5 (22.9 kDa) and α6 (33 kDa) proteasome subunits in brain lysates isolated from the indicated control (irs*1* KO +/+) and IRS1-depleted mice (*irs1* KO -/-). The total protein load was used as a control for equal protein loading. 100% has been arbitrarily set to the average values of each set of control samples. All error bars show SEM.

**
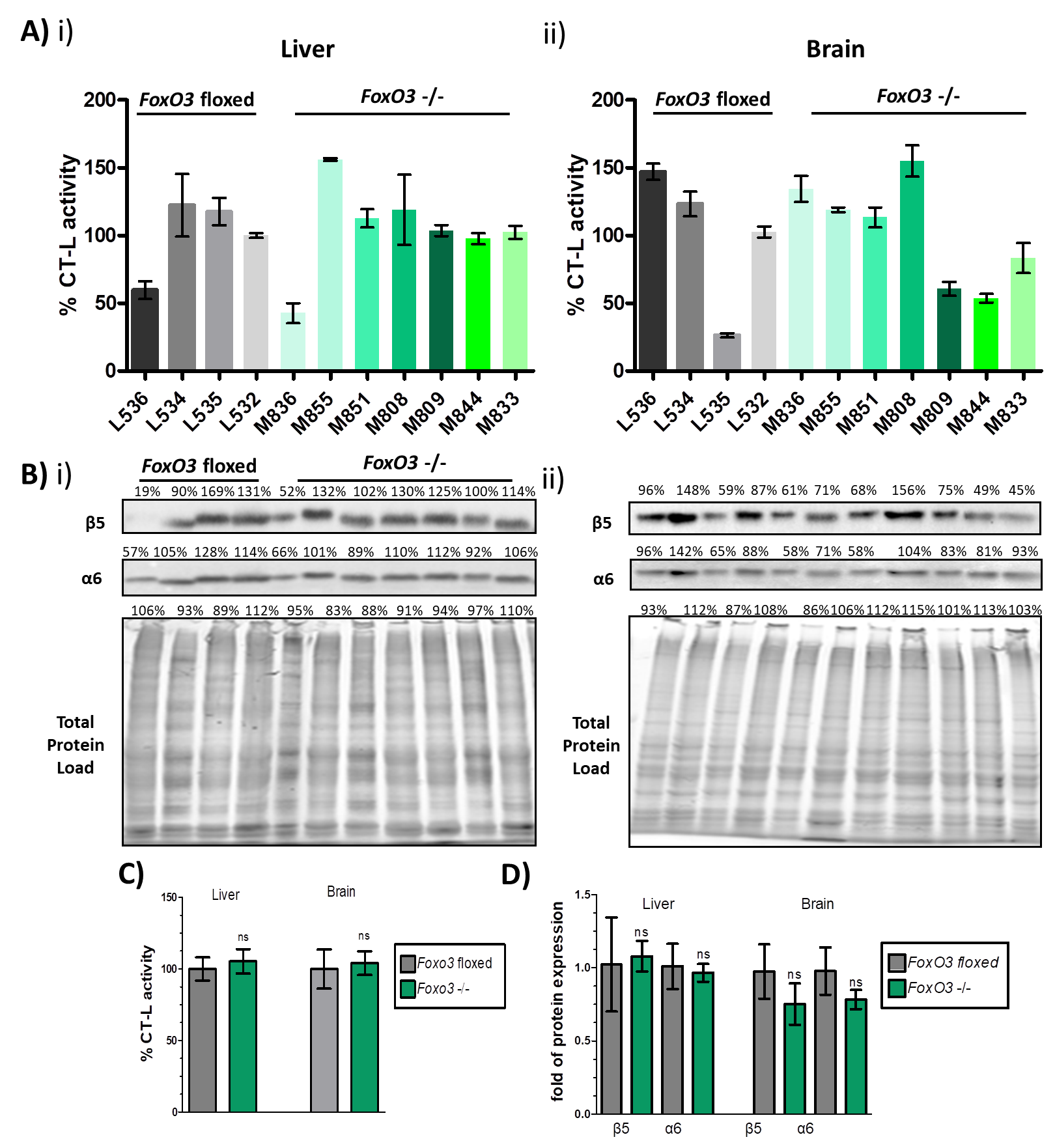
**

**Figure S6.** ***Foxo3* KO does not affect proteasome activity in liver and brain: (**A) % CT-L activities and (B) Immunoblot analysis of β5 (22.9 kDa) and α6 (33 kDa) proteasome subunits in i) liver and ii) brain lysates isolated from the indicated control (FoxO3 floxed) and FoxO3-depleted mice (*foxo3* KO -/-). C) mean % CT-L activities and D) mean values immunoblot analysis of the indicated proteasome subunits in the liver and brain of the indicated control (FoxO3 floxed) and FoxO3-depleted mice. The total protein load was used as a control for equal protein loading. 100% or 1 has been arbitrarily set to the average values of each set of control samples. All error bars show SEM. *Foxo3* floxed n=4, *Foxo3* -/- n=7.

| Primer | Sequence (5’ -> 3’) |
| --- | --- |
| FOXO1-ChIP psmb6 (β1) fwd | CACGACCTGACCATTCCAGT |
| FOXO1-ChIP psmb6 (β1) rev | ATTTGCCTCACCAGAGGCTT |
| FOXO1 ChIP PSMB7 (β2) fwd | GGTGGAGGACTCCCAACATT |
| FOXO1 ChIP PSMB7 (β2) rev | CTGCTCCGGAAAACGAACTG |
| FOXO1 ChIP PSMB5 (β5) fwd | GAAGGGTTGCTGTATGGCGA |
| FOXO1 ChIP PSMB5 (β5) rev | TTGTTAACGCGCCTGAGGTT |
| FOXO1 ChIP PSMB4 (β7) fwd | GGGAATTCCTCCACTGCCTC |
| FOXO1 ChIP PSMB4 (β7) rev | CTCAGAGCCGGCCAATAGAG |
| PSMB5 (β5) transcript fwd | GCTGGCTAACATGGTGTATCAT |
| PSMB5 (β5) transcript rev | AAGTCAGCTCATTGTCACTGG |
| PSMA6 (α6) transcript fwd | AGGCTATTAACCAGGGTGGACTT |
| PSMA6 (α6) transcript rev | GTGAGTCACTGTGCTGGAATCC |
| GAPDH transcript fwd | CATCACTGCCACCCAGAAGACTG |
| GAPGH transcript rev | ATGCCAGTGAGCTTCCCGTTCAG |

**Table S1.**  **Primer pairs used for RT-PCR analysis**

| **ID** | **Promoter sequence cloned into the LightSwitch™ Promoter Reporter Vector** |
| --- | --- |
| pβ5-lightswitch | ACACACACACACAAATTAGCCTGTTGTGGAGGCAGGCGCCTGTAATCCCAGCTACTCGGGAGGCTGAGGCAGGAGAATCGCTTGAACCTGGGAAGCAGAGGTTGCAGTGAGCAGAGATCGTGCCACTGTGTTCCAGCCTGGGGGACACAGCAAGACTTGGTCTCAAAAAACAAACAAACAAACAAACAAAAACAATACAGCATATGCAGAGTCTGCTGGGGGAAAGATGGGGCCGAACTGGGCATCAGAGGAAAGTGGAATTTCGGTGTTGATAGATGTTGCTGCTAGAATCCAACAAGGAAGGCCAGAGACTGACTTCATAGAACCATAAAGCAGGAGGCAACATTTCATACATATGCACACTTCCCGGTTTGTTATAGTTCCCAAAAGATTTGAAAAATGAAGGTGGGTGGAGGGAGGAAGAGGCAATGAAATATAGTATGTTCCTGAATAAAAGGCATCATCTTAGCAAGTTGAAGTTAGAGAATCTCTTGGTACATATTTATGCAGTCTCAACCGTCCTCCCGTCTCTGAGATGGTAAATTACTACTGACACTTATTCAGGTCTGAACTGAGAATCTAGTTTGTGGATTAACTGGACCCTTTGCTTTTTCTTCTTCCATCTGCCTTTTAGGCAATTCATTACTTATTCGAATTTCCACCAGAAGGTAGGAGTCAAGAGAACGAATATTGGATATTAATAACCGGTTACTGTTAAAGAAGGGTTGCTGTATGGCGACTGACAACAAGAGGTATAGCACTT**TTATTTTG**TTGCGTTGTTTTACCACTAATTGCTGTGGTTTAACTAATATAGGGAAATGGCCTCAGAATGGATCATTATTTTACCCAATTTTAGAAGGTGCCCTTAAACAAGATGGCAACCTCAGGCGCGTTAACAAGGTGCTGAAGCAGCAGCGATGTAACCTGTTGCTTGGACACCAGGCTGGAGTGGGAGGACTCACCGCTAAGGGTTCAGAACCGATTTTCAACCAGTCACGCC |
| mut-pβ5-lightswitch | ACACACACACACAAATTAGCCTGTTGTGGAGGCAGGCGCCTGTAATCCCAGCTACTCGGGAGGCTGAGGCAGGAGAATCGCTTGAACCTGGGAAGCAGAGGTTGCAGTGAGCAGAGATCGTGCCACTGTGTTCCAGCCTGGGGGACACAGCAAGACTTGGTCTCAAAAAACAAACAAACAAACAAACAAAAACAATACAGCATATGCAGAGTCTGCTGGGGGAAAGATGGGGCCGAACTGGGCATCAGAGGAAAGTGGAATTTCGGTGTTGATAGATGTTGCTGCTAGAATCCAACAAGGAAGGCCAGAGACTGACTTCATAGAACCATAAAGCAGGAGGCAACATTTCATACATATGCACACTTCCCGGTTTGTTATAGTTCCCAAAAGATTTGAAAAATGAAGGTGGGTGGAGGGAGGAAGAGGCAATGAAATATAGTATGTTCCTGAATAAAAGGCATCATCTTAGCAAGTTGAAGTTAGAGAATCTCTTGGTACATATTTATGCAGTCTCAACCGTCCTCCCGTCTCTGAGATGGTAAATTACTACTGACACTTATTCAGGTCTGAACTGAGAATCTAGTTTGTGGATTAACTGGACCCTTTGCTTTTTCTTCTTCCATCTGCCTTTTAGGCAATTCATTACTTATTCGAATTTCCACCAGAAGGTAGGAGTCAAGAGAACGAATATTGGATATTAATAACCGGTTACTGTTAAAGAAGGGTTGCTGTATGGCGACTGACAACAAGAGGTATAGCACTT**GGACCCCT**TTGCGTTGTTTTACCACTAATTGCTGTGGTTTAACTAATATAGGGAAATGGCCTCAGAATGGATCATTATTTTACCCAATTTTAGAAGGTGCCCTTAAACAAGATGGCAACCTCAGGCGCGTTAACAAGGTGCTGAAGCAGCAGCGATGTAACCTGTTGCTTGGACACCAGGCTGGAGTGGGAGGACTCACCGCTAAGGGTTCAGAACCGATTTTCAACCAGTCACGCC |

**Table S2.**  **Promoter sequences cloned into the LightSwitch™ Promoter Reporter Vector to drive luciferase expression.** The detected IRE at -237 and the mutagenized version are highlighted in Bold.
